# Organ Donation after Damage Control Strategy in Trauma Patients: Experience from First Level Trauma Center in Italy

**DOI:** 10.3390/life12020214

**Published:** 2022-01-30

**Authors:** Michele Altomare, Shir Sara Bekhor, Stefano Piero Bernardo Cioffi, Marco Sacchi, Federica Renzi, Andrea Spota, Roberto Bini, Federico Ambrogi, Federico Pozzi, Arturo Chieregato, Osvaldo Chiara, Stefania Cimbanassi

**Affiliations:** 1 General Surgery and Trauma Team, ASST Niguarda, Milano, Piazza Ospedale Maggiore 3, 20162 Milan, Italy; michele.altomare@ospedaleniguarda.it (M.A.); stefanopierocioffi@ospedaleniguarda.it (S.P.B.C.); federica.renzi@ospedaleniguarda.it (F.R.); andrea.spota@ospedaleniguarda.it (A.S.); roberto.bini@ospedaleniguarda.it (R.B.); osvaldo.chiara@ospedaleniguarda.it (O.C.); 2 Department of Surgical Sciences, Sapienza University of Rome, Piazzale Aldo Moro 5, 00185 Rome, Italy; 3 Department of Medical-Surgical Physiopathology and Transplantation, University of Milan, Festa del Perdono 7, 20122 Milan, Italy; shir.bekhor@studenti.unimi.it; 4 Department EMERGENZA URGENZA-E.A.S. SOREU Metropolitana, 20161 Milan, Italy; marco.sacchi@ospedaleniguarda.it; 5 Department of Clinical Science and Community Health, University of Milan, Festa del Perdono 7, 20122 Milan, Italy; federico.ambrogi@unimi.it; 6 Neurointensive Care Unit, Grande Ospedale Metropolitano Niguarda, 20162 Milan, Italy; federico.pozzi@ospedaleniguarda.it (F.P.); arturo.chieregato@ospedaleniguarda.it (A.C.)

**Keywords:** trauma, organ donation, trauma donors, damage control surgery, damage control

## Abstract

Background: Organ donation (OD) remains the only therapeutic option for end-stage disease in some cases. Unfortunately, the gap between donors and recipients is still substantial. Trauma patients represent a potential yet underestimated pool of organ donors. In this article, we present our data on OD after damage control strategy (DCS). Materials and Methods: A retrospective, observational cohort study was conducted through a complete revision of data of consecutive adult trauma patients (>18 years old) who underwent OD after DCS between January 2018 and May 2021. Four subgroups were created [Liver (Li), Lungs (Lu), Heart (H), Kidneys (K)] to compare variables between those who donated the organ of interest and those who did not. Results: Thirty-six patients underwent OD after DCS. Six patients (16.7%) were excluded: 2(5.6%) for missing data about admission; 4(11.1%) didn’t receive DCS. Mean ISS was 47.2 (SD ± 17.4). Number of donated organs was 113 with an organs/patient ratio of 3.8. The functional response rate was 91.2%. Ten organs (8.8%) had primary nonfunction after transplantation: 2/15 hearts (13.3%), 1/28 livers (3.6%), 4/53 kidneys (7.5%) and 3/5 pancreases (60%). No lung primary nonfunction were registered. Complete results of subgroup analysis are reported in supplementary materials. Conclusion: Organ donation should be considered a possible outcome in any trauma patient. Aggressive damage control strategy doesn’t affect the functional response rate of transplanted organs.

## 1. Introduction

### 1.1. Organ Procurement after Damage Control Resuscitation Strategies

The notion that an individual may improve or preserve the life of another by receiving his tissue has captured our collective imagination. The extraordinary pace of scientific and technological advancement of the 20th century has made organ transplantation a reality. Unfortunately, the gap between donors and recipients has always been significant worldwide. 

Trauma patients represent an underestimated potential pool of organ donors. In the last decades the improvement of resuscitative strategies, including surgical and non-surgical procedures, has allowed trauma surgeons to increase the success rate of these strategies in trauma patients. Unfortunately, failure to rescue is a well-known outcome for trauma and acute care surgeons [1,2,3]. In real-life experience, it is often difficult to focus the attention on possible outcomes not related to the patient’s survival. Costs and ethical implications of aggressive resuscitative strategies on in extremis patients often delay the decision-making process in the emergency department. On the other hand, recent studies show that trauma donors (TDs) are younger, have fewer comorbidities compared to non-traumatic donors (NTDs) and produce more organs/donor (3.5 vs. 2.4) [4]. Moreover, TDs are more likely to yield an extrarenal organ and exhibit lower (better) Kidney Donor Risk Index scores, a predictor of graft longevity [5]. Unfortunately, the proportion of TDs has significantly decreased from 55.3% in 1987 to 35.8% in 2016 [6]. These data could be related to better outcomes for trauma patients due to the improved quality of resuscitative treatments in first-level trauma centers [7]. It is also important not to underestimate the risk of a “soft” approach in patients considered in extremis. In fact, when a patient’s life cannot be saved, the short survival time should be considered useful in saving another human life awaiting organs. Therefore, an “aggressive” approach could increase the number of lost organs suitable for donation. A recent review proposed by Ackerman et al. concluded that given the vital role TDs play in meeting the demand for organs, trauma surgeons are important stewards of this resource. Unfortunately, the literature does not have reliable data about the European attitude on organ donation after the implementation of damage control strategies (DCSs) in trauma patients. Predictors for organ donation in NTDs are well known [8], while variables related to trauma and DCS, both in the emergency department and intensive care unit, are still debated [9,10]. 

For these reasons, as a first-level trauma center in Italy with more than 800 major trauma patients referred per year, we aim to analyze our data on TDs after DCS to evaluate the role of an aggressive approach towards critical patients and the potential presence of factors associated with organ donation.

### 1.2. Aims

The primary endpoint of this study is to describe our data regarding TDs after DCS. Therefore, we analyzed variables related to each harvested organ to identify factors associated with organ procurement after DCS. The secondary endpoint is the evaluation of short-term outcomes of organ transplantation in terms of primary nonfunction rate. 

## 2. Material and Methods

A retrospective, observational cohort study was conducted through a complete data review of consecutive adult trauma patients (>18 years old) who underwent organ donation (OD) after DCS between January 2018 and May 2021. Inclusion criteria were the following: at least one procured and transplanted organ; at least one surgical or not surgical (i.e., massive transfusion protocol, MTP) procedure performed during trauma management; availability of all records regarding emergency department (ED) admission, DCS management, and transplant. Four subgroups were created (Liver (Li), Lungs (Lu), Heart (H), Kidneys (K)) to compare variables between those who donated the organ of interest and those who did not. We further analyzed the impact on organ donation of the following variables: Injury Severity Score (ISS) at admission; the number of DCS procedures (nDCS) performed; Vasoactive Usage (VaU); Organ-Specific Injury (OSI); activation of MTP (ED-MTP); the total number of blood products administered. In the H group, we also analyzed variables related to patients’ stability at arrival in the ED (i.e., systolic blood pressure (SBP), heart rate (HR), lactate level (LAC), and base excess (BE)). Variables under evaluation were selected according to the currently available literature. The population was divided into two groups according to the organ outcome. The grade of organ injury was classified according to the Abbreviated Injury Scale (AIS) 2015 revision. Lung injuries were not analyzed because the presence of parenchyma contusion excludes organs from donation. Short-term outcomes of transplanted organs were evaluated in terms of primary nonfunction and functional response rates. All donors included in the analysis are death brain donors (DBDs). Nowadays, In the Italian and European experience, the pool of death cardiac donors (DCDs) are almost all non-trauma related. Cardiac arrest in trauma patients often leads to aggressive treatment that hardly preserves the physiological reserve. All organ procurements were performed following the standard procedure. Intern protocol for intensive care unit management was applied to all trauma donors (supplementary Material). 

Mann–Whitney–Wilcoxon and the Fisher–Freeman–Halton tests analyzed the association between categorical variables. No multivariable models were built considering the low power of the enrolled population. Results with *p* < 0.05 were considered statistically significant.

## 3. Results

### 3.1. Study Population

Thirty-six patients underwent organ procurement following trauma during the study period. Six patients (16.7%) were excluded from the initial cohort; two patients (5.6%) were excluded on account of missing data regarding emergency department admission, four patients (11.1%) did not receive any DCS procedure. Thus, 30 patients were enrolled in the study for 40 months at a rate of 0.75 patients per month. The mean age was 45.2 (SD ± 20.9) with a male/female ratio of 2.75. The mean Charlson Comorbidity Index was 0.97 (SD ± 1.2). Twenty-nine patients (96.6%) had blunt trauma, and the remaining case was a penetrating trauma. Clinical parameters and demographic characteristics, pre-hospital rescue, and ED shock room (SR) evaluation are summarized in Table 1, Table 2 and Table 3, respectively.

The total number of donated organs was 113, with an organs/patient ratio of 3.8. Fifteen patients (50%) donated the heart, and 28 (93.3%) donated the liver, including four split organs procurement. Among 27 (90.0%) kidney donors, one patient gave only the right kidney due to chronic kidney disease and multiple parapelvic cysts of the left one. Five patients (16.6%) donated the pancreas for transplantation. Mean ISS at admission was 47.2 (SD ± 17.4). Four patients (13.3%) had a liver injury that did not preclude organ donation; two patients had AIS II liver injury, one patient had AIS III and one had liver AIS IV. Two patients (6.6%) had kidney injuries of AIS II and III respectively and they donated both right and left kidneys. No heart injuries were registered. Only four patients (13.3%) were eligible for lung donation because of the gravity of lung injuries related to trauma and invasive mechanical ventilation. The mean lung injury AIS was 2.4 for patients who did not donate the lung and 1.7 for lung donors (*p* = 0.47).

Twenty-eight patients (93.3%) were intubated on the scene and referred with a 3T GCS. Two patients arrived in the SR with GCS 3 and a Guedel airway mask because of a failed endotracheal intubation (EI).

Mean SBP and HR at presentation were 111.9 (SD ± 39.2) and 99.9 (SD ± 33.9), respectively. The mean shock index at admission was 1.0 (SD ± 0.52). ABG at admission showed a mean pH of 7.1 (SD ± 0.23), mean BE of 9.9 (SD ± 8.54), and mean lactate level of 6.24 (SD ± 4.73). Two patients (6.6%) received bilateral thoracostomies and 19 (63.3%) were treated with vasoactive drugs during PH resuscitation maneuvers or in SR. Fifty-one DCS procedures were performed with a procedure per patient ratio of 1.7 (SD ± 1.9). Eleven procedures (21.5%) were performed in the ED, including five instances of extraperitoneal pelvic packing (EPP), five bilateral thoracostomies, and one patient was treated with REBOA positioning in SR. Forty DCS procedures were performed in the operating room or hybrid angiography/operating room, including: six damage control laparotomies; three damage control thoracotomies; 16 damage control craniotomies; eight limb external fixations; one decompressive laparotomy; and six endovascular embolizations with angiography. In 25 cases (83.3%) the massive transfusion protocol (MTP) was activated. Details on MTP and the use of vasoactive drugs in ICU are reported in supplementary materials. Intensive Care Unit (ICU) mean length of stay was 3.4 (SD ± 2.6). All patients underwent methylprednisolone hormone replacement therapy (15 mg/kg) and were given levothyroxine (150 mcg) to control trauma-related pituitary failure. Therapies (clonidine and/or esmolol) directed to protect the heart during the catecholaminergic storm related to the Cushing reflexes phase were applied. Desmopressin acetate was also used in patients affected by diabetes insipidus. Only one (3.3%) patient needed renal replacement therapy during ICU recovery.

### 3.2. Short-Term Outcomes of Transplanted Organs and Subgroup Analysis

The functional response rate was 91.2%. Ten organs (8.8%) had primary nonfunction after transplantation: 2/15 hearts (13.3%), 1/28 livers (3.6%), 4/53 kidneys (7.5%) and 3/5 pancreases (60%). No lung primary nonfunction were registered. In groups H, Li and Lu no significant differences were found regarding: ISS (H 49.3 ± 14.1 vs. 44.9 ± 18.9 *p* = 0.3938; Li 47.5 ± 9.5 vs. 47.1 ± 17.2 *p* = 0.9667; Lu 42.5 ± 11.4 vs. 47.8 ± 17.4 *p* = 0.6908); nDCS (H 1.27 ± 0.9 vs. 2.07 ± 2.435 *p* = 0.6789; Li 1.5 ± 0.5 vs. 1.7 ± 1.9 *p* = 0.549; Lu 1.1 ± 0.1 vs. 1.7 ± 2.02 *p* = 0.7098); VaU (H 60% vs. 66.7%; Li 50.0% vs. 64.3%; Lu 75.0% vs. 65.1%); OSI (Li 0.0% vs. 14.3%). For group H, also SBP (112.07 ± 30.479 vs. 111.67 ± 45.133 *p* = 0.56), HR (101.47 ± 39.960 vs. 98.33 ± 24.773 *p* = 0.6183), BE (−9.11 ± 8.779 vs. −10.69 ± 7.917 *p* = 0.5897) and lactate level (5.39 ± 3.930 vs. 7.08 ± 5.138 *p* = 0.4067) were evaluated without differences between donors and non-donors. In groups K, VaU and nDCS, procedures were found to significantly increase organ procurement (73.9% vs. 0.0% *p* = 0.0328; 1.8 ± 1.9 vs. 0.3 ± 0.4 *p* = 0.0334). No 30-day mortality related to graft disfunction was registered in our series. Complete results of the subgroup analysis are reported in the Supplementary Materials.

## 4. Discussion

Severely injured trauma patients are a global concern since injury is responsible for over five million deaths each year [11]. The prevalence of blunt and penetrating trauma in Italy has increased over the last decades, bringing a new era for acute care and trauma surgery in our country [7]. The creation and implementation of trauma network with hub-and-spoke models in Italy is having a good impact on the mortality and morbidity of trauma patients. Unfortunately, traumatic brain injury (TBI) remains a leading cause of death in trauma patients and, in the case of severe brain injury, the mortality rate is still high [12]. In the past 30 years, the management of trauma patients has changed dramatically [13]. During this period, the organ donor population has also changed. In an effort to deal with the demand, the list of criteria for accepting organs for donation has been expanded. Donation after cardiac death (DCD) and the Expanded Criteria Donors permit harvesting of organs that may have been previously discarded and are now routinely utilized [14,15,16]. The interaction between these two epidemiological shifts was examined in two recent systematic reviews published in the *Journal of Trauma and Acute Care Surgery* [4,5]. Trauma Donors seem to be younger and with fewer comorbidities compared to NTDs. Our study population supports this data with an average age of 45 years and a mean Charlson Comorbidity Index of 0.97. These two demographic characteristics surely impact the higher organ procurement rate of TDs described in the literature (3.7 vs. 2.4) and confirmed in our study (3.8). Moreover, our study highlights good outcomes of transplanted organs from TDs, as demonstrated by the low primary nonfunction rate. These findings may be explained by the natural selection of healthier donors related to trauma epidemiology. However, compared to non-trauma patients, procurement from TDs may be more challenging due to the difficulty of controlling bleeding and maintaining hemodynamic stability following brain death due to acute blood loss. Successful recovery and organ harvesting require careful attention to the potential donor, even after resuscitation and declaration of brain death. In our experience, an aggressive approach to critical and in extremis patients seems to improve the OD rate, especially for kidneys, without compromising the transplanted organs. This result supports the literature demonstrating that kidneys from TDs have a better Kidney Donor Risk Index (KDRI) compared to NTDs. On the other hand, the small sample size and a low number of events in the groups under examination may have influenced our results, suggesting the possibility of publication bias. 

A systematic review proposed by Cameron et al. [5] examined factors associated with organ donation. Of 27 studies included in the analysis, the effect of various factors on OD was examined in six studies, among which three used multivariable logistic regression. In their review, the only clinical factor found positively related to organ donation was the use of thyroid hormone replacement therapy. In addition, cardiopulmonary resuscitation in the emergency department and multiple injuries seem to reduce OD in eligible TDs. According to the latest guidelines [17], all patients in our study underwent hormone replacement therapy. It appears that liver and kidney organ-specific injuries do not affect OD or outcomes of the transplanted organ. Moreover, despite the high mean ISS score at admission, all patients in our series donated at least one organ. Lung injury is a relative contraindication to OD due to direct trauma, resuscitation fluid overload, shock lung, early pneumonia, lung injury due to mechanical ventilation. In our series, almost all lungs were excluded from the donation. Therefore, organ injury should be considered independently for each organ under evaluation and should not exclude a priori a potential donation. 

Interestingly, vasoactive usage in a pre-hospital setting/emergency department and a number of surgical and non-surgical procedures of DCS performed seem to significantly improve the possibility of kidney procurement in our series. Taking into account the possible bias related to the limited sample size, this finding suggests that an aggressive damage control strategy approach might not affect the functional response rate of transplanted organs in terms of PNF and 30-day mortality related to graft dysfunction. Indeed, hemodynamic stability allows good perfusion of organs suitable for transplantation and could facilitate the procurement of those organs that better withstand prolonged times of ischemia. Both liver and kidneys should always be considered possible organs for donation when evaluating trauma patients referred to a trauma center. 

This study has several limitations: the small sample size and the absence of a control group could have affected the comparison between groups. On the other hand, the present cohort is one of the biggest reported in European literature during the last years. The retrospective nature of the study could have affected data entry, but the ethical implications of this issue could complicate the design of a prospective clinical trial. 

## 5. Conclusions

Considering the results of this study, the role of surgical and non-surgical resuscitation strategies in offering a pool of potential organ donors should be further examined in future prospective studies. The authors truly believe that given the vital role TDs play in meeting the demand for organs, trauma surgeons are important stewards of this resource and should always consider the possibility of organ donation while evaluating and resuscitating even the most severely injured patients.

## Figures and Tables

**Table 1:** Demographic Characteristics.

Patient ID	SEX	AGE	BMI	CCI *	Intentionality	Mechanism of Trauma
1	M	31	21.10	0	N	Blunt
2	F	62	31.20	2	N	Blunt
3	M	46	22.50	0	N	Blunt
4	M	60	24.80	2	N	Blunt
5	M	59	23.40	1	N	Blunt
6	F	79	29.10	3	N	Blunt
7	M	34	22.90	0	N	Blunt
8	M	79	24.60	3	N	Blunt
9	M	15	21.50	0	N	Blunt
10	M	57	27.50	1	N	Blunt
11	F	32	18.40	0	N	Blunt
12	M	69	27.00	2	N	Blunt
13	M	18	22.40	0	N	Blunt
14	M	76	24.00	5	N	Blunt
15	F	66	20.50	2	N	Blunt
16	M	26	27.70	0	N	Blunt
17	M	59	26.10	1	Y	Blunt
18	M	67	23.40	2	N	Blunt
19	F	51	22.00	1	Y	Penetrating
20	F	31	22.50	0	N	Blunt
21	M	15	19.00	0	Y	Blunt
22	M	18	27.30	0	N	Blunt
23	M	42	26.30	0	N	Blunt
24	F	44	21.50	0	N	Blunt
25	M	24	24.90	0	N	Blunt
26	M	22	22.90	0	N	Blunt
27	M	65	26.10	2	N	Blunt
28	M	61	26.10	2	N	Blunt
29	F	23	23.00	0	N	Blunt
30	M	25	23.10	0	N	Blunt

* Charlton Comorbidity Index; Body mass index.

**Table 2:** Pre-hospital rescue data (PH).

Patient ID	SBP *	HR °	GCS ^#^	N ° Caridac Arrest	Vasoactive	Time-Prehosp to TC ** (MIN)	EI ^§^
1	100	100	3	0	N	17	Y
2	150	80	6	0	N	n/a	Y
3	110	80	3	0	N	n/a	Y
4	140	85	10	0	N	27	N
5	160	180	15	0	N	35	Y
6	120	80	7	0	N	n/a	Y
7	60	90	3	0	Y	n/a	Y
8	180	120	3	0	N	n/a	Y
9	88	134	3	1	Y	n/a	Y
10	n/a	80	3	0	N	n/a	Y
11	116	65	3	0	N	n/a	Y
12	0	0	3	1	Y	n/a	Y
13	100	80	3	1	Y	n/a	Y
14	80	80	3	0	N	n/a	Y
15	120	120	3	0	N	70	Y
16	70	130	6	1	N	50	Y
17	140	165	3	0	N	160	N
18	150	118	3	1	Y	60	Y
19	50	120	4	1	Y	180	Y
20	50	120	3	0	N	70	Y
21	60	55	3	2	Y	n/a	Y
22	50	120	3	0	Y	60	Y
23	120	70	4	0	N	n/a	Y
24	64	n/a	3	4	N	n/a	Y
25	140	80	5	0	N	50	Y
26	60	140	3	0	N	70	Y
27	160	80	5	0	N	n/a	Y
28	0	0	3	1	Y	85	Y
29	0	0	3	2	Y	70	Y
30	0	0	3	2	Y	n/a	Y

* Systolic blood pressure; ° Heart rate; ^#^ Glasgow Coma Scale; ^§^ Endotracheal Intubation; ** Trauma Center.

**Table 3:** SR presentation data.

Patient ID	Cardiac Arrest Admission	Time to ROSC (MIN)	SBP *	HR °	GCS ^#^	Shock Class	Shock Index	PH	BE ^$^	LAC ^£^
1	N		85	140	3T	3	1.65	7.2	−5	3.2
2	N		150	90	3T	2	0.60	7.3	−5.5	1.5
3	N		136	70	3T	2	0.51	7.2	−7	4.2
4	N		194	69	3	2	0.36	7.3	−3.6	2.08
5	N		140	113	3T	2	0.81	7.3	2	1.99
6	N		180	74	3T	2	0.41	7.4	0	2.2
7	N		70	90	3T	3	1.29	7.1	−10	9.7
8	N		180	126	3T	2	0.70	7.2	2.6	1.8
9	N		104	126	3T	3	1.21	6.94	−14	7.6
10	N		156	80	3T	1	0.51	6.9	−17	4.4
11	N		110	100	3T	2	0.91	7	−14	5
12	N		40	75	3T	4	1.88	7.1	−11.9	5.9
13	N		112	91	3T	3	0.81	7,4	1.6	1.1
14	N		80	80	3T	3	1.00	7.2	−8.9	6.3
15	N		125	98	3T	1	0.78	7.3	−4.7	3
16	Y	18	40	0	3T	4	0	6.5	−29	15.4
17	N		130	180	3	2	1.38	6.8	−16.8	8.5
18	N		70	120	3T	3	1.71	6.9	−14.5	1.1
19	Y	15	120	61	3T	1	0.51	7	−19.3	14.6
20	N		100	122	3T	2	1.22	7	−13.8	8.96
21	Y	5	100	150	3T	2	1.50	7.1	−15.2	16.4
22	N		60	134	3T	4	2.23	7	−16	6
23	N		134	98	3T	2	0.73	7.3	4.8	1.4
24	Y	5	90	100	3T	3	1.11	7.3	−6.9	7.9
25	N		120	80	3T	1	0.67	7.2	−9.2	7.9
26	N		109	140	3T	4	1.28	7.3	−6.4	3.5
27	N		101	73	3T	2	0.72	7.3	−3.2	2.6
28	N		100	110	3T	2	1.10	7.3	−7.2	4.9
29	Y	20	70	120	3T	4	1.71	6.6	−30	17
30	N		150	87	3T	2	0.58	6.8	−19	11

* Systolic blood pressure; ° Heart rate; ^#^ Glasgow Coma Scale; ^$^ Base Excess; ^£^ Lactate.

## Data Availability

The data presented in this study are available on request from the corresponding author. The data are not publicly available to preserve confidentiality.

## Supplementary Materials

The following are available online at https://www.mdpi.com/article/10.3390/life12020214/s1. File S1: Complete results of subgroup analysis.
