# Supplementary material for: Organ Donation after Damage Control Strategy in Trauma Patients: Experience from First Level Trauma Center in Italy
Source: Life (Basel). 2022 Jan 30;12(2):214. doi: 10.3390/life12020214 (PMC8877798; doi:10.3390/life12020214)
Supplement: Supplementary file 1 [file life-12-00214-s001.zip › life-1522136-supplementary.pdf]

SUPPLEMENTARY MATERIALS

Table S1. Comparison of HEART DONORS.

VARIABLE	HEART (YES) N = 15	HEART (NO) N = 15	*p*-VALUE
ED-SBP ADMISSION	112.07 ± 30.479 (range: 40–150)	111.67 ± 45.133 (range: 40–194)	*p* = 0.5609 [a]
ED-HR	101.47 ± 39.960 (range: 0–180)	98.33 ± 24.773 (range: 61–150)	*p* = 0.6183 [a]
ED-BE	-9.11 ± 8.779 (range: -29–4.8)	-10.69 ± 7.917 (range: -30–2.6)	*p* = 0.5897 [a]
ED-LAC	5.39 ± 3.930 (range: 1.1–15.4)	7.08 ± 5.138 (range: 1.1–17)	*p* = 0.4067 [a]
ED-MTP	Yes 14 (93.3%) No 1 (6.7%)	Yes 11 (73.3%) No 4 (26.7%)	*p* = 0.32950 [b]
N° RBC	11.00 ± 9.121 (range: 2–40)	16.07 ± 14.364 (range: 2–50)	*p* = 0.3165 [a]
VASOACTIVE PH/ED	Yes 9 (60.0%) No 6 (40.0%)	Yes 10 (66.7%) No 5 (33.3%)	*p* = 1 [b]
NUMBER OF PROCEDURES	1.27 ± 0.998 (range: 0–4)	2.07 ± 2.435 (range: 0–8)	*p* = 0.6789 [a]
ISS	49.33 ± 14.108 (range: 25–75)	44.93 ± 18.954 (range: 17–75)	*p* = 0.3938 [a]

[a]. Mann–Whitney non-parametric test for unpaired samples; [b]. Two-tailed Fisher's exact test for categorical data.

Table S2. Comparison of KIDNEYS DONORS.

VARIABLE	KIDNEY (YES) N = 27	KIDNEY (NO) N = 3	*p*-VALUE
ED-MTP	Yes 24 (88.9%) No 3 (11.1%)	Yes 2 (66.7%) No 1 (33.3%)	*p* = 0.37055 [a]
N° RBC	14.38 ± 12.821 (range: 2–50)	10.00 ± 4.899 (range: 4–16)	*p* = 0.7457 [b]
VASOACTIVE PH/ED	Yes 20 (74.1%) No 7 (25.9%)	Yes 0 (0.0%) No 3 (100.0%)	*p* = 0.03284 * [a]
NUMBER OF PROCEDURES	1.88 ± 1.948 (range: 0–8)	0.33 ± 0.471 (range: 0–1)	*p* = 0.0334 * [b]
ISS	48.27 ± 17.353 (range: 17–75)	43.33 ± 9.741 (range: 35–57)	*p* = 0.615 [b]
KIDNEY INJURY (Y/N)	Yes 2 (7.4%) No 25 (92.6%)	Yes 0 (0.0%) No 3 (100.0%)	*p* = 1 [a]

*$p < 0.05$; [a]. Two-tailed Fisher's exact test for categorical data; [b]. Mann–Whitney non-parametric test for unpaired samples.

Table S3. Comparison of LIVER DONORS.

VARIABLE	LIVER (NO) N = 2	LIVER (YES) N = 28	*p*-VALUE
ED-MTP	Yes 2 (100.0%) No 0 (0.0%)	Yes 23 (82.1%) No 5 (17.9%)	*p* = 1 [a]
N° RBC	16.00 ± 5 (range: 11–21)	13.36 ± 12.638 (range: 2–50)	*p* = 0.2415 [b]
VASOACTIVE PH/ED	Yes 1 (50.0%) No 1 (50.0%)	Yes 18 (64.3%) No 10 (35.7%)	*p* = 1.00000 [a]
NUMBER OF PROCEDURES	1.50 ± 0.5 (range: 1–2)	1.68 ± 1.965 (range: 0–8)	*p* = 0.549 [b]

ISS	47.50 ± 9.5 (range: 38–57)	47.11 ± 17.257 (range: 17–75)	$p = 0.9667$ [b]
LIVER INJURY (Y/N)	Yes 0 (0.0%) No 2 (100.0%)	Yes 4 (14.3%) No 24 (85.7%)	$p = 1$ [a]

[a]. Two-tailed Fisher's exact test for categorical data; [b]. Mann–Whitney non-parametric test for unpaired samples.

Table S4. Comparison of LUNGS DONORS.

VARIABLE	LUNG (YES) N = 4	LUNGS (NO) N = 26	*p*-VALUE
ED-MTP (1-Y/0-N)	Yes 3 (75.0%) No 1 (25.0%)	Yes 22 (84.6%) No 4 (15.4%)	$p = 0.53841$ [a]
N° RBC	5.75 ± 3.031 (range: 2–10)	14.73 ± 12.739 (range: 2–50)	$p = 0.0701$ [b]
VASOACTIVE PH/ED	Yes 3 (75.0%) No 1 (25.0%)	Yes 16 (61.5%) No 10 (38.5%)	$p = 1$ [a]
NUMBER OF PROCEDURES	1.00 ± 0 (range: 1–1)	1.77 ± 2.025 (range: 0–8)	$p = 0.7098$ [b]
ISS	42.50 ± 11.435 (range: 25–57)	47.85 ± 17.428 (range: 17–75)	$p = 0.6908$ [b]
LUNG INJURIES (Y/N)	N/A	N/A	N/A

[a]. Two-tailed Fisher's exact test for categorical data; [b]. Mann–Whitney non-parametric test for unpaired samples.

ICU Protocols

Brain Death Donor Management in the Intensive Care Unit

Brain death causes a marked physiological alteration in many organ systems, especially in the cardiorespiratory and endocrine systems, and in association with the consequences of the trauma poses a particular challenge in the management of these patients. Our approach is a cardiorespiratory and endocrine resuscitation, tailored to the physiopathological characteristics of the patients. The target of our protocol, from a hemodynamic point of view, is to maintain organ perfusion (MAP >70, urine output >0.7 ml/kg/h) through a correction of hypovolemia and judicious use of vasopressors and inotropes, with the help of a multimodal advanced monitoring with PICCO (Pulse Index Continuous Cardiac Output), SVO2 and lactate. We use a protective ventilator strategy with a Tidal Volume 6–8 ml/kg, keeping a Plateau Pressure <28, Driving Pressure <15, PEEP 5–10 cm H20, and with the use of recruitment maneuvers. All patients receive methylprednisolone 15 mg/kg, thyroid hormones supplementation and desmopressin in case of a diagnosis of diabetes insipidus. Our goal in these cases is to maintain euvolemia and keep sodium 135–150 mEq/l. In patients with trauma-related coagulopathy, we correct with the guidance of thromboelastogram. Red blood cells are transfused to keep the hemoglobin level 10 gr/dl.

Table S5. Organ injury: ISS—Injury Severity Score, AIS—Abbreviated Injury Scale.

Patient ID	ISS	AIS Brain	AIS Liver	AIS Kidney	AIS Lung	AIS Pancreas	AIS Extremities
1	57	5	N/A	N/A	2	N/A	N/A
2	34	5	N/A	N/A	0	N/A	N/A
3	43	5	N/A	N/A	3	N/A	N/A
4	26	5	N/A	N/A	0	N/A	1
5	38	5	N/A	N/A	3	N/A	N/A
6	29	5	N/A	N/A	2	N/A	N/A
7	75	6	N/A	N/A	0	N/A	N/A

8	45	5	N/A	N/A	4	N/A	1
9	42	5	N/A	N/A	4	N/A	1
10	57	4	N/A	N/A	3	N/A	5
11	50	4	3	N/A	5	N/A	2
12	35	5	N/A	N/A	3	N/A	N/A
13	35	5	N/A	3	3	N/A	N/A
14	35	5	N/A	N/A	2	N/A	N/A
15	38	5	N/A	N/A	3	N/A	3
16	59	N/A	N/A	N/A	5	N/A	5
17	50	4	N/A	N/A	5	N/A	3
18	33	5	N/A	N/A	2	N/A	N/A
19	75	6	N/A	N/A	0	N/A	N/A
20	66	5	2	N/A	4	2	5
21	66	5	0	0	4	N/A	3
22	75	6	N/A	2	3	N/A	2
23	38	5	N/A	N/A	0	N/A	3
24	17	N/A	4	N/A	2	N/A	N/A
25	25	5	N/A	N/A	0	N/A	N/A
26	43	5	N/A	N/A	0	N/A	N/A
27	75	6	N/A	N/A	2	N/A	N/A
28	20	N/A	N/A	N/A	2	N/A	N/A
29	57	N/A	2	N/A	4	3	4
30	66	N/A	N/A	N/A	4	2	3

Table S6. Transplanted organs.

Patient ID	Heart	Lungs	Liver	Hemiliver	Kidney	Pancreas	Number of Donated Organs
1	1.00	2.00	1.00	0.00	0.00	0.00	4.00
2	1.00	0.00	1.00	0.00	2.00	0.00	4.00
3	1.00	0.00	1.00	0.00	2.00	1.00	5.00
4	0.00	0.00	1.00	0.00	2.00	0.00	3.00
5	1.00	0.00	0.00	0.00	2.00	0.00	3.00
6	0.00	0.00	1.00	0.00	1.00	0.00	2.00
7	0.00	0.00	1.00	0.00	2.00	0.00	3.00
8	0.00	0.00	1.00	0.00	2.00	0.00	3.00
9	1.00	0.00	1.00	1.00	2.00	1.00	6.00
10	0.00	0.00	0.00	0.00	2.00	1.00	3.00
11	1.00	0.00	1.00	0.00	2.00	0.00	4.00
12	0.00	0.00	1.00	0.00	2.00	0.00	3.00
13	1.00	2.00	1.00	1.00	2.00	1.00	8.00
14	0.00	0.00	1.00	0.00	0.00	0.00	1.00
15	0.00	0.00	1.00	0.00	0.00	0.00	1.00
16	1.00	0.00	1.00	0.00	2.00	0.00	4.00
17	1.00	0.00	1.00	0.00	2.00	0.00	4.00
18	0.00	0.00	1.00	0.00	2.00	0.00	3.00
19	0.00	0.00	1.00	0.00	2.00	0.00	3.00
20	0.00	0.00	1.00	0.00	2.00	0.00	3.00
21	0.00	0.00	1.00	0.00	2.00	0.00	3.00
22	1.00	0.00	1.00	0.00	2.00	0.00	4.00
23	1.00	1.00	1.00	0.00	2.00	1.00	6.00
24	0.00	0.00	1.00	0.00	2.00	0.00	3.00
25	1.00	2.00	1.00	1.00	2.00	1.00	8.00
26	1.00	2.00	1.00	0.00	2.00	0.00	6.00
27	1.00	0.00	1.00	0.00	2.00	0.00	4.00
28	0.00	0.00	1.00	0.00	2.00	0.00	3.00
29	0.00	0.00	1.00	0.00	2.00	0.00	3.00
30	1.00	0.00	1.00	0.00	2.00	0.00	4.00
